# Antimicrobial sensitivity profile and bacterial isolates among suspected pyogenic meningitis patients attending at Hawassa University Hospital: Cross-sectional study

**DOI:** 10.1186/s12866-020-01808-5

**Published:** 2020-05-19

**Authors:** Demissie Assegu Fenta, Kinfe Lemma, Henok Tadele, Birkneh Tadesse Tilahun, Birrie Derese

**Affiliations:** 1 School of Medical Laboratory Science, Hawassa University College of Medicine and Health Sciences, Hawassa, Ethiopia; 2 Department of Internal Medicine, Hawassa University College of Medicine and Health Sciences, Hawassa, Ethiopia; 3 Department of Pediatrics, Hawassa University College of Medicine and Health Sciences, Hawassa, Ethiopia; 4 Department of Neurology, Hawassa University College of Medicine and Health Sciences, Hawassa, Ethiopia

**Keywords:** Pyogenic meningitis, Bacterial isolates, Culture, Antimicrobial sensitivity, Hawassa, Ethiopia

## Abstract

**Background:**

Bacterial meningitis is a serious inflammation of the meninges. Antimicrobial therapy on early cerebrospinal fluid (CSF) examination has an important role in diagnosis. The disease is still challenging in developing countries because of poor (diagnostic set-up, socioeconomic conditions, management), and misuse of antimicrobial therapy results in emerging antimicrobial-resistant strains. Therefore, this hospital based cross sectional study was aimed to assess the antimicrobial sensitivity profile and bacterial isolates among patients suspected of pyogenic meningitis at Hawassa University Hospital from February 2017 to 2018.

**Results:**

A total of 394 patients suspected as meningitis were included. Of these 210 (53.3%) were males and 184 (46.7%) were females. The carriage rate of bacterial pathogens was 27(6.9%). The common clinical presentations were fever 330 (83.8%), headache 205 (52.0%) and neck stiffness 179(45.4%) followed by altered mental status 125(31.7%). Neck stiffness *P* = 0.001 (AOR = 1.18, 95% CI 1.06–6.53), Hx of seizure *P* = 0.043, (AOR = 1.39, 95% CI 1.15–5.99), Nuchal rigidity *P* = 0.001* (AOR = 1.26, 95% CI 1.06–4.48) were significantly associated with culture positivity.

The pathogens isolated in this study were *N. meningitidis* the most frequent isolate 12(44.4%) followed by *S. pneumoniae* 5 (18.5%), *E. coli* 4(14.8%), *H. influenza* 3(13.6%), *S. aureus* 2(11.1%) and *K. pneumoniae* 1(3.7%). *S. pneumoniae* was (100%) resistance to penicillin, (80%) amoxicillin, and (20%) Cefotaxime. *S. aureus* was (100%) resistant to penicillin, amoxicillin, and ciprofloxacin. *N. meningitidis* was (100%) resistant to penicillin, (66.7%) Ceftriaxone and (41.7%) chloramphenicol. In this study a single isolate was also resistant to a different antibiotic.

**Conclusion:**

The prevention of bacterial meningitis needs serious attention since the isolated bacteria showed single and multiple antimicrobial susceptibility patterns and the variable nature of isolated etiological agents makes it reasonable to provide continuous future updates on local resistance of common antibiotics and optimize the most frequent bacteria associated with meningitis in the hospital. Therefore; further, survey study with a better design of antimicrobial susceptibility at large scale to control the spread of antibiotic-resistant bacteria and the change in the causative organism of bacterial meningitis in the study area and at a national level is required.

## Background

Bacterial meningitis is still a major public health threat in developing countries. Its management is also still a priority of public health because of its rapid onset and high level of morbidity and mortality [1]. It is an overwhelming infection with high morbidity and mortality worldwide. It is responsible for approximately 170,000 deaths annually [1, 2]. Meningitis continues to remain among the leading causes of childhood death, in areas with high HIV prevalent where, the mortality can be as high as 50% particularly in the developing countries [3, 4]. Nowadays in spite of the great improvement in modern antibiotics and healthcare services, the life-threatening problem of bacterial meningitis is not eliminated (4). This could be due to delayed presentation, treatment initiation [5], limited diagnostic facility, and poor standards of care are some of the major reasons for poor treatment outcome of meningitis in Africa [6]. For these reasons, the management of patients with suspected meningitis presents an exceptional challenge to physicians working in resource-poor settings [3, 6].

Pyogenic meningitis is an infection of the membranes and cerebrospinal fluid (CSF) surrounding the brain and spinal cord caused by a large variety of bacteria, viruses, fungi, and parasites [7]. The infection caused by bacteria is quite severe resulting in brain damage, hearing loss, learning disability and death if not treated early. Patients with bacterial meningitis often present with onset of at least one of the following four symptoms: fever, headache, nuchal rigidity, and altered mental status. However, the sensitivity of the classic triad of fever, nuchal rigidity, and change in mental status is low in the diagnosis of adult bacterial meningitis [8].

One of the greatest challenges of meningitis is misdiagnosis from other causes of meningitis such as cryptococcal meningitis, viral meningitis and parasitic meningitis [9].

Rapid, accurate, and confirmatory microbiological diagnosis and immediate treatment of meningitis causative agents are necessary because of its potentially devastating effects [10]. Gram staining is widely available and as an inexpensive and rapid procedure routinely performed for primary diagnosis of bacterial meningitis [11]. However, gram staining sensitivity is not reliable, so CSF culture is mandatory to diagnose bacterial meningitis [11]. Implementation of rational antimicrobial therapy based on the findings of early CSF examination has an important role in the limitation of bacterial meningitis and also its future outbreaks. Consequently, appropriate treatment options are also difficult to access and the management of suspected cases is mainly clinical and empiric because of limited access and lack of availability of materials and trained personnel [6]. This situation is more pronounced in the meningitis belt of Africa where several pyogenic meningitis outbreaks have been documented [12, 13].

The epidemiology of bacterial meningitis varies by geographic region and by factors such as the availability and use of vaccines, and other risk factors associated with the immune status of individuals, age, HIV/AIDS, cancer, and other immune depression agents. Geographically the highest-burden of meningococcal meningitis occurs in sub- Saharan Africa countries with estimated cases of 300 million population [14]. Ethiopia is one of the countries in the region where bacterial meningitis is a common problem even with a recent outbreak in the southern region [15].

From all cases of bacterial meningitis 80% is caused by *N. meningitidis*, *S. pneumoniae*, and *H. influenza* [16]. All age groups can be affected by meningococcus, but over two-thirds of all cases of bacterial meningitis occur in children less than 5 years old and adolescents. More than 80% of meningitis is caused by *H. influenza* in children less than 5 years old. Group B hemolytic streptococcus (*S. agalactae*) is a common cause of meningitis in neonates, *E. coli* is a frequent cause of meningitis in neonates and is a rare cause after infancy [16].

Bacterial antimicrobial resistance is a worldwide problem particularly, in developing countries like Ethiopia, the situation is serious [17]. Despite advancements in antimicrobial therapy, and vaccine availability the mortality remains high, in developing countries among adults [18] and children [19]. However, increasing the frequency of reports of bacterial resistance in vitro to the commonly used drugs has raised a concern that this choice of management may not be longer appropriate [20, 21]. Because of the absence of well-organized laboratories, inadequate distribution and availability of vaccination, unavailability of antimicrobial agents and lack of proper surveillance on bacterial meningitis and antimicrobial resistance continue to be a great challenge in Ethiopia [22].

The existence of drug-resistant bacteria and the ever-increasing number of drug-resistant strains with time is becoming a great threat to the population [23, 24]. Local data generation on the isolation and antimicrobial sensitivity profile of the pathogens is an urgent issue to decide health priorities, to allocate resources, and planning the effective use of antimicrobial drugs and vaccines in the region. There are few studies regarding the prevalence of common bacterial isolates causing the disease and their antibiotic sensitivity pattern. Furthermore, no uniformity in the practice among clinicians and health facilities to manage the disease [25, 26]. Therefore, this study was aimed to assess the antimicrobial sensitivity profile and bacterial isolates among patients suspected of pyogenic meningitis at Hawassa University Hospital, Southern Ethiopia.

## Result

### Socio-demographic characteristics and study participants

A total of 394 patients who were suspected as cases of pyogenic meningitis in the hospital were included in this study. Out of these 210 (53.3%) were males and 184 (46.7%) were females with the age ranged of 3 months to 75 years with a mean age of 14.6 years. The majority of the participants 174(44.2%) were found within the age range of 14–50 years, 135(34.3%) within the age range of 5–14 years followed by 72(18.3%) less than 5 years and 13(3.3%) of them above 50 years.

From a total of 394 suspected patients, 117(29.7%) showed typical clinical characteristics of bacterial meningitis. But, only 27(6.9%) patients had proven clinical and laboratory evidence for bacterial meningitis by the identification of a causative pathogen in the CSF sample in this study.

### CSF characteristics

Lumbar puncture was performed to collect CSF from 394 patients. The overall bacterial carriage rate isolated in this study was 27(6.9%). The carriage rate was higher in males 16(4.1%) than females 11(2.8%). Similarly, It was higher in children of age less than 14 years old 15(55.6%) than adults greater than 14 years of age 12(44.4%).

Among CSF samples, 196 (49.7%) were collected after the first dose of antibiotic administration and 108 (27.4%) after 24 h of antibiotic treatment and 90(22.8%) were collected before the administration of antibiotics. From a total, CSF sample collected 158 (40.2%) were visibly turbid in appearance during collection.

The median white cell count in the CSF was 682 cells/μl with the median differential count of 67 and 33% turbid samples had neutrophils and lymphocytes respectively. Whereas the median CSF protein value was 105 mg/dl and that of glucose value was 42 mg/dl. Patients with proven bacterial meningitis and unidentified etiology had higher CSF cell count, higher CSF protein and lower glucose ratio as compared with suspected patients and have no bacterial growth on culture (Table 1).

**Table 1:** Cerebrospinal fluid characteristics of the study participants

Parameters	Frequency			
	Yes (%)	No (%)	Unknown (%)	*P*-Value
Prior antibiotic treatment	90 (22.8)	304 (77.2)	–	0.001*
Indian ink Positive	13 (3.4)	381 (96.6)	–	
AFB positive	20 (5.1)	374 (94.9)	–	**0.013**
Gram stain	24 (6.1)	370 (93.9)	–	
HIV	20 (5.1)	279 (70.8)	95 (24.1)	**0.034**
White blood cells (Median)	682 cells/mm^3^	–	–	
Neutrophil (Median)	67%	–	–	
Lymphocyte (Median)	33%	–	–	
Glucose (Median)	42 mg/dl			0.672
Protein (Median)	105 mg/dl			
Hospital stay (Median days)	7.5 days			0.734

*AFB* Acid Fast Bacilli, *HIV* Human immunodeficiency Virus, *mg* milligram, *dl* deciliter, *mm*^*3*^ millimeter cub

### Clinical characteristics

The most common clinical presentations of patients in this study were fever 330 (83.8%), headache 205 (52.0%) and neck stiffness 179(45.4%) followed by altered mental status upon arrival 125(31.7%). Less common symptoms included vomiting 71(18%), photophobia 54(13.7%) and seizures 30(7.6%). Majority 340 (86.3%) of patients who visited the hospital with an average of 7.5 days after the onset of symptoms and some of them had one or more clinical features consistent with critical illness including severe wasting.

### Culture and biochemical characteristics

From a total of 394 CSF samples, 27(6.9%) revealed a causative organism by culture. Among these, 7 were positive by gram’s stain and also showed culture positivity. On the other hand from turbid CSF samples which were not positive by culture 20(12.6%) samples were AFB positive and 13 (8.2%) of them were Indian ink positive and the rest 125(79.1%) samples were unidentifiable by culture.

From bacterial pathogens isolated and proven by culture, 20 (74.1%) of the isolates were gram-negative organisms while 7(25.9%) were gram-positive organisms. *N. meningitidis* was found to be the most frequent isolate 12(44.4%) followed by *S. pneumoniae* 5 (18.5%), *E. coli* 4(14.8%), *H. influenza* 3(13.6%), *S. aureus* 2(11.1%) and *K.pneumoniae* 1(3.7%). The majority of N. meningitidis 6 (50%) were isolated from adults of age greater than 14 years. However, in this study, multiple infections or isolates were not observed (Table 2).

**Table 2:** Distribution of bacterial isolates by sex and age from culture-positive samples at Hawassa University, Hospital (*n* = 27)

Bacterial isolate	Frequency	Patient category and isolate frequency				
		Male	Female	Children (3mth-)		Adults (> 14 yrs)
				3mth-5 yrs	> 5 yrs- 14 yr	
N.meningitidis	12 (44.4%)	8	4	4	2	6
H.influenzae	3 (13.6%)	2	1	0	1	2
E.coli	4 (14.8%)	3	1	1	1	2
S.pneumoniae	5 (18.5%)	2	3	2	1	2
K.pneumoniae	1 (3.7%)	1	0	1	0	0
*S. aureus*	2 (11.1%)	0	2	1	0	1
Total	27 (100%)					

*E.coli Escherchia coli, N.meningitidis Nesseria meningitides, S.pneumoniae Streptococcus pneumoniae, K.pneumoniae Klebsella pneumoniae*

Organisms that were isolated from culture media were subjected to commonly used antibiotics for susceptibility testing in the hospital. The results of the antibiotic susceptibility pattern showed the following trend (Table 3).

**Table 3:** Antibiotic susceptibility pattern of bacterial isolates from CSF in patients with bacterial meningitis, Hawassa University Hospital

Antibiotic Susceptibility Pattern of Gram negative organisms (% resistant)												
	*N.meningitidis*			*H.influenza*			*E.coli*			*K.pneumoniae*		
	S	I	R	S	I	R	S	I	R	S	I	R
Penicillin G	–	–	Mic> 0.6012 (100%)	–	–	3 (100%)	–	1 (25%)	3 (75%)	–	–	1 (100%)
CAF	1 (8.3%)	6 (50%)	5 (41.7%)	–	1 (33.3%)	2 (66.7%)	1 (25%)	2 (50%)	1 (25%)	–	1 (100%)	–
Co trimoxazole	3 (25%)	5 (41.7%)	4 (33.3%)	1 (33.3%)	–	2 (66.7%)	–	2 (50%)	2 (50%)	–	–	1 (100%)
Ciprofloxacin	5 (41.7%)	5 (41.7%)	2 (16.7%)	2 (66.7%)	–	1 (33.3%)	1 (25%)	1 (25%)	2 (50%)	–	1 (100%)	–
Ceftriaxone	4 (33.3%)	0	8 (66.7%)	2 (66.7%)	1 (33.3%)	–	1 (25%)	1 (25%)	2 (50%)	–	–	1 (100%)
Cefotaxime	5 (41.7%))	0	7 (58.3%	2 (66.7%)	–	1 (33.3%)	1 (25%)	1 (25%)	2 (50%)	–	–	1 (100%)
Gentamycin	8 (66.7%)	3 (25%)	1 (8.3%)	2 (66.7%)	1 (33.3%)	–	2 (50%)	1 (25%)	1 (25%)	1 (100%)	–	–
Antibiotic Susceptibility Pattern of Gram positive organisms (% resistant)												
	*S.pneumoniae*			*S. aureus*								
	S	I	R	S	I	R	S	I	R	S	I	R
Penicillin Mic> 0.06	0	0	5 (100%)	–	–	2 (100%)						
Amoxicillin	–	1 (20%)	4 (80%)	–	–	2 (100%)						
Ciprofloxacin	–	4 (80%)	1 (20%)	–	–	2 (100%)						
Cefotaxime	2 (40%)	2 (40%)	1 (20%)	–	–	2 (100%)						
Ceftriaxone	2 (40%)	3 (60%)	–	–	2 (100%)	–						
Erythromycin	2 (40%)	0	0	–	–	2 (100%)						
Gentamicin	2 (40%)	1 (20%)	2 (40%)	–	2 (40%)	3 (60%)						
Vancomycin MIC(0.5-2 μg)	5 (100%)	–	–	2 (100%)	–	–						
CAF	–	2 (40%)	3 (60%)	–	2 (100%)	–						

*S* Sensitive, *R* Resistant, *I* Intermiadate, *E.coli Escherchia Coli, N.meningitidis Nesseria meningitides*, *S.pneumoniae Streptococcus pneumoniae*, *K.pneumoniae Klebsellapneumoniae*, *Pen* Penicillin, *Amp* Ampicillin, *CAF* Chloramphenicol, (0) Not done, (−) Not Sensitive, Intermiadate, resistance

Among gram-positive organisms, *S. pneumonia* showed a total drug resistance against penicillin (100%), amoxicillin. (80%), Cefotaxime (20%) and *S. aureus* were found to be (100%) resistant to penicillin, amoxicillin, and ciprofloxacin. Whereas from gram-negative bacteria, *N. meningitidis* was (100%) resistant to penicillin, 66.7% for Ceftriaxone and 41.7% to chloramphenicol (Table 3).

The test revealed high-level resistance to antibiotics used mainly as standard regimens for empiric treatment of bacterial meningitis in the study area. Resistance to different antibiotics by a single isolate was also found (Table 3).

Bivariate and multivariate logistic regression was to assess the association of culture positivity and clinical diagnosis of bacterial meningitis. Based on this (Sex) being male was significantly associated with culture positivity and clinical diagnosis of bacterial meningitis *P* = 049, (AOR = 1.37, 95% CI 1.31–7.02) (Table 4). The clinical features such as Neck stiffness *P* = 0.001 (AOR = 1.18, 95% CI 1.06–6.53, Hx of seizure *P* = 0.043, (AOR = 1.39, 95% CI 1.15–5.99), Nuchal rigidity *P* = 0.001* (AOR = 1.26, 95% CI 1.06–4.48 and other clinical signs showed a significant association of culture positivity and clinical diagnosis (Table 4).

**Table 4:** Association of culture positivity and clinical characteristics with bacterial meningitis, at Hawassa University, Hospital

Clinical characteristics		Total N(%)	Culture negative N(%)	Culture Positive N(%)	Odds ratio(95%CI)	*P* value
Sex	Male	210 (53.3)	194 (92.4)	16 (7.6)	1.37 (1.3–7.02)	0.049
	Female	184 (46.7)	173 (94.1)	11 (5.9)		0.074
Age	< 5 yr	71 (18.0)	60 (84.5)	11 (15.5)	–	–
	5- < 14 yr	162 (41.1)	155 (95.6)	7 (4.4)		
	14–50	141 (35.7)	133 (94.0)	8 (6.0)		
	> 50 years	20 (5.2)	19 (95.0)	1 (5.0)		
Fever	Yes	330 (83.8)	306 (92.7)	24 (7.3)	1.2 (1.35–4.0)	0.784
	No	64 (16.2)	61 (95.3)	3 (4.7)		
Head ache	Yes	205 (52.1)	187 (91.2)	18 (9.8)	0.71	0.469
	No	189 (47.9)	180 (95.0)	9 (5.0)		
Neck Stiffness	Yes	179 (45.4)	157 (87.7)	22 (14.0)	1.18 (1.06–6.53)	0.001
	No	215 (54.6)	210 (97.7)	5 (2.3)		
Hx of seizure	Yes	30 (7.6)	13 (43.4)	17 (56.6)	1.39 (1.15–5.99)	0.043
	No	364 (92.4)	354 (97.3)	10 (3.3)		
Duration of illness before presentation	< 3 days	84 (21.3)	76 (90.5)	8 (10.5)	–	0.137
	3-7 days	175 (44.4)	161 (92)	14 (8.0)		
	> 7 days	135 (34.3)	130 (96.2)	5 (3.8)		
Temp.Grade	High	74 (18.8)	70 (94.3)	4 (5.7)	–	0.543
	Low	115 (29.2)	101 (87.8)	14 (13.8)		
	Normal	205 (52)	196 (95.6)	9 (4.4)		
Nuchal rigidity	Yes	172 (43.6)	151 (87.8)	21 (13.2)	1.26 (1.06–4.48)	0.001*
	No	222 (56.3)	216 (97.3)	6 (2.7)		
Brudniski’s Sign	Yes	88 (22.3)	72 (81.8)	16 (22.2)	2.19 (2.07–5.51)	0.001
	No	306 (77.6)	295 (96.4)	11 (3.6)		
Kernig’s sign	Yes	67 (17.0)	54 (80.6)	13 (19.4)	1.15 (1.09–2.73)	0.008
	No	327 (82.9)	313 (95.7)	14 (4.3)		
Glasgow coma scale	Normal	259 (65.7)	242 (93.0)	17 (7.0)	–	0.523
Impairedconsciousn		128 (32.5)	119 (93.0)	9 (7.0)		
Coma		7 (1.8)	6 (85.7)	1 (15.3)		
Prehospitalantibiotic	Yes	202 (51.3)	194 (96.0)	8 (4.0)	0.54 (0.211.39)	0.198
	No	192 (48.7)	173 (90.0)	19 (10)		
Inhospital preLP abc	Yes	252 (64.0)	246 (97.6)	6 (2.4)	1.16 (1.14–2.95)	0.015
	No	142 (36)	121 (85.2)	21 (14.8)		
CSF appearance	Clear	324 (82.2)	321 (99.1)	3 (0.9)		0.000
	Turbid	47 (11.9)	23 (95.8)	24 (4.2)		
	Bloody	23 (5.9)	23 (100)	–		
CSF cell level	< 10	259 (65.7)	257 (99.2)	2 (0.8)	–	0.001*
	10–100	64 (16.3)	53 (82.8)	11 (17.2)		
	> 100	71 (17.9)	57 (80.0)	14 (20)		
CSF pro category	< 46	273 (69.2)	270 (98.8)	3 (1.2)		0.001*
	46–100	71 (18.0)	56 (78.9)	15 (21.1)		
	> 100	50 (12.8)	41 (82.0)	9 (18)		
CSF glucose level	< 40	125 (31.7)	104 (83.2)	21 (16.8)	1.29 (1.11–3.77)	0.010
	> 40	269 (68.3)	263 (97.7)	6 (2.3)		

*CSF* Cerebrospinal Fluid, *Tem.* Temperature, *NO* Number, *abc* antibiotics, *CSF pro* CSF protein, *LP* Lumbar punctur

## Discussion

Despite the availability of effective antibiotics, bacterial meningitis is still a major cause of morbidity and mortality in low-income countries like Ethiopia. Acute bacterial meningitis is a medical emergency, which warrants early diagnosis and aggressive therapy. Most often therapy for bacterial meningitis in low-income countries is empirical. The successful management of meningitis depends upon the identification of the types of organisms that causes the disease and the selection of an effective antibiotic against the organism in question [27, 28]. Thus, the data presented in this study could provide information to take immediate public health actions and to clinicians in the study area on the selection of the antimicrobial agent and to avoid inconsistent management of the patient.

From a total of 394 meningitis, suspected cases in this study 210(53.3%) and 184(46.7%) were males, and females respectively. Infection with bacterial meningitis was significantly associated with males than females, (AOR = 1.37, 95% CI (1.3–7.02, *P* = 0.049). Similar findings were reported from research conducted in India [29]. The majority of isolated bacteria were found to be within the age range of fewer than 14 years old. A consistent finding was reported from India [29]. However, different finding to our study was also reported from the same country India [30].

From a total of 394 patients where CSF samples were analyzed and the causative bacterium was identified in only 27 samples which had an isolation rate of 27(6.9%). This result was comparable to the previous two studies conducted in Gondar, where an isolation rate was 5.2, 5.6% [22, 31], Southern Ethiopia (6.6%) [32] and Hawassa Ethiopia (6.4%) [33]. However, this finding was higher than the studies reported from another study in Gondar [34] and Saudi Arabia (0.3%) [35]. On the other hand, this finding was lower than studies done in Uganda (44.12%) [36] and two different studies conducted in Indian colleges (14.37, 62.5%) [37, 38]. The reason for low yield of bacterial isolates on culture in this study could be due to clinical misdiagnosis of meningitis cases, lumbar puncture might have been done unnecessarily, non-availability of special media for specific pathogen isolation in the laboratory setting, fastidious nature of organisms, antibiotic treatment before lumbar puncture and the difference in study population.

The most common clinical presentation in the current study was fever 330(83.8%), headache 205(52.1%) and neck stiffness 179(45.4) followed by altered mental status upon arrival 125(31.6%). Inconsistent findings to this study were also reported from Saudi Arabia fever (80%) altered mental status (70.0%) followed by neck rigidity (41.7%) [39]. This difference could be due to the difference in clinician management, geographical area or difference in the guideline used in meningitis management.

Isolated bacteria’s from CSF in the current study were, *N. meningitidis* 12(44.4%)*, S. pneumoniae* 5(18.5%)*, E. coli* 4 (14.8%)*, H. influenza* 3(13.6%)*, S. aureus* 2(11.1%) and *K.pneumoniae* 1(3.7%). Inconsistent findings to this study were reported from *Malawi* [4] Gondar [34, 40] and Nigeria [41]*.* The predominant organism in our study was found to be *N. meningitidis.* However, in agreement findings were reported from other studies from Nigeria [41] and Iran [39] indicating that *E. coli* was the predominant bacteria next to *S. pneumoniae*. In another study from a systematic review and meta-analysis conducted in different regions of Europe [28], *Haemophilus influenzae* accounted (35.5%), was the predominant organism next to *Streptococcus pneumoniae* (19.6%) and other pathogens (12%). This difference might be due to the difference in the study population, study design, sample size and geographic area of the study population.

In this study, bacterial isolates were subjected to different antibiotics to observe their susceptibility patterns for each organism (Table 3). Accordingly, penicillin was found to be 100% resistant for both gram-negative and gram-positive bacteria. Among gram-negative bacteria, *N.meningitidis* was 41.7% resistant to chloramphenicol, 66.7% ceftriaxone and 33.3% to cotrimoxazole. Comparable findings were reported from Gondar [31]. Whereas among gram-positive bacteria. *S. pneumoniae* was found to be 80% resistant to amoxicillin and 20% to ciprofloxacin and 40% intermediate to ceftriaxone (Table 3). Similar findings were reported from the Paraná State United States [42] indicating that *S. pneumoniae* was resistant to amoxicillin, penicillin G, ceftriaxone, ciprofloxacin, and Vancomycin. This could be due to an ever-increasing empirical treatment of meningitis and other infections by this antimicrobial agent.

In our study *E.coli* was resistance to Penicillin (75%), chloramphenicol (25%), Ceftriaxone (50%), Cefotaxime (50%), Ciprofloxacin (50%) and co-trimoxazole (50%) (Table 3). In contrast to this study the antibiotic resistant *E.coli* was reported for penicillin (12%), Ceftriaxone (44%), Co-trimoxazole (52%), chloramphenicol (52%) [28] and ceftriaxone (86.7%), Ciprofloxacin (40%), and gentamycin (26.7%) from India [29].

In the present study, S. *aureus* also showed a high level of drug resistance against amoxicillin (100%), penicillin G. (100%), ciprofloxacin (100%), and erythromycin (100%). Comparable findings were reported from Gondar Ethiopia [18] and Iran [21].

On the other hand, there is an exceedingly high rate of resistance of microorganisms to two or more antibiotic resistance (Multidrug resistance) including commonly prescribed third-generation cephalosporin (ceftriaxone of Cefotaxime) in the current study. Similar findings were reported from Gondar [40] and Hawassa [33]. This could be due to empiric antibiotic treatment use, overutilization of antibiotics and indiscriminate use of antibiotics in developing countries and the study area. No bacterial isolates were resistant to Vancomycin in this study, being this drug is indicated as a therapeutic option in the case of multidrug-resistant bacteria, together with the cephalosporin drugs. Comparable results were reported from Gondar [31] Hawassa [33] and India [30].

More than half of the patients in the current study have a long duration of illness and empirical antibiotic treatment before hospital presentation, resulting in a poor outcome of patients with bacterial meningitis which depends on a timely selection of appropriate antibiotics. This result was different from studies conducted in Gondar, Ethiopia [22], Denmark [23] and review study from [24] which showed a positive relation of short duration illness and timely selection of antibiotics. This difference might be the difference in the use of standard guidelines for clinical presentation and lack of culture for etiologic identification and antibiotics susceptibility testing at all levels.

As ongoing effort is made to prevent and control bacterial meningitis and the condition seeks greater emphasis more than ever before and appropriate measures to be acted immediately. Therefore, evidence-based management of bacterial meningitis by using culture and antimicrobial susceptibility tests should be strengthened before empirical treatment. Moreover, the recommended or routinely prescribed drugs of choices in the study area like ciprofloxacin and ceftriaxone are found to decrease their susceptibility in all of the bacterial isolates in this study. And further study with a better design, large sample size and survey of antimicrobial susceptibility at large scale should be done to draw important information.

The prevention and control of bacterial meningitis need serious attention since the isolated bacteria showed high single and multiple antibiotic-resistant pathogens. Therefore, the variable nature of isolated etiological agents and antimicrobial susceptibility patterns of bacterial meningitis made it reasonable to provide continuous future updates on local resistance patterns of the most frequent bacteria associated with meningitis and optimize institutional infection control policies.

## Conclusion

The present study revealed *Neisseria meningitides*, *S. pneumoniae*, *Haemophilus influenza*, *E.coli, K.pneumoniae*, and *S. aureus* were the most common bacterial etiologic agents isolated from CSF culture The prevention and control of bacterial meningitis need serious attention since the isolated bacteria showed high single and multiple antibiotic-resistant pathogens. This could be a reflection of the inappropriate use of antibiotics before and after hospital presentation, unavailability of a guideline regarding the selection of drugs and clinical diagnosis and lack of well-organized laboratory infrastructure. Therefore, the variable nature of isolated etiological agents and antimicrobial susceptibility patterns of bacterial meningitis made it reasonable to provide continuous future updates on local resistance patterns of the most frequent bacteria associated with meningitis in combating drug resistance problem and optimize institutional diagnostic methods in the study area.

Further, a survey study with a better design of antimicrobial susceptibility at large scale to control the spread of antibiotic-resistant bacteria and the change in the causative organism of bacterial meningitis in the study area and at a national level. Additionally, ways to improve the yield of diagnostic methods need to be considered at each level of the health service area.

## Methods

### Study design, area and period

A hospital-based cross-sectional study design was conducted from February 2017–2018 at Hawassa University Hospital to assess antimicrobial sensitivity profile and bacterial isolates among suspected pyogenic meningitis patients. It is the largest referral hospital in the region with 450 beds and serving as a teaching specialized hospital with a catchment population of over 18 million. It is also an institution where specialized clinical services that are not available in other public or private health institutions are provided to the whole region in the Southern, Ethiopia.

### Source population

All patients above 3 months of age who were seen at emergency, outpatient departments, and admission wards of pediatrics and internal medicine, and suspected of pyogenic meningitis were included. Those who were HIV and TB- positive and not volunteer to give consent and assent were excluded from this study.

### Sampling technique and sample size

Three hundred ninety-four CSF samples were collected systematically from suspected patients in the study hospital in the study period. Patients that met the case definition of meningitis or compatible clinical manifestations (fever, headache, and neck stiffness) by the physician and obtained from the nursing desk were included in the study. Between 5 and 8 samples were collected per week by using the laboratory register as a sampling frame from the last one- year data.

### Specimen collection and processing

Sociodemographic and clinical data were collected by using a standard questionnaire. HIV results and antiretroviral therapy (ART) status were obtained from their follow-up chart by using a checklist.

Cerebrospinal fluid (CSF) specimens were collected aseptically by a lumbar puncture at the discretion of the attending physicians and transferred into sterile tubes. The specimens were sent to the microbiology laboratory within half an hour of collection. Conventional bacteriological methods were employed to isolate and identify the bacteria. Cerebrospinal fluid specimens were seeded on to culture media, blood agar, chocolate agar, and MacConkey agar plates (Oxoid Ltd., Basingstoke, and Hampshire, UK) was prepared as per the manufacturer’s instruction and incubated at 35–37 °C aerobically for 48 h in humid air plus 5–10% CO2 for chocolate agar plates by placing them in a candle jar, which provide a concentration to create a microaerophilic condition for fastidious bacteria.

The CSF samples were also used for total and differential cell count, glucose and protein determination, gram and AFB stain and Indian ink preparation for *Cryptococcus neoformans*. Cultured plates, which did not show any growth, were further incubated for an additional 24 h to observe the presence of slow-growing bacteria. The identification of organisms on culture was performed by morphological colony characteristics, staining, biochemical and serological tests.

Antibiotic sensitivity test was conducted on pure culture isolates employing the disc diffusion method [43, 44] for the commonly used antibiotics: gentamicin (10 μg), Penicillin G (10 IU), chloramphenicol (30Fg), ciprofloxacin (5Fg), Ceftriaxone (30 μg), Cefotaxime (30 μg), Erythromycin (15 μg), Norfloxacin (10 μg), Amoxicillin (30 μg), Vancomycin (30 μg) and cotrimoxazole (25Fg) (Oxoid Ltd). The diameters of growth inhibition around the discs were measured by using a ruler and interpreted as sensitive, intermediate or resistant as per the CLSI guideline (Clinical laboratory Standard index) document [45, 46]. Reference strains: *E. coli* (ATCC 25922), *Staphylococcus aureus* (ATCC 24923*), Streptococcus pyogenes* (ATCC 19615), and *Streptococcus agalactiae* (ATCC12403) were used as controls according to the National Committee for Clinical Laboratory Standards (NCCLS) [42, 47]. Protein, glucose and cell count (Total and differential) were also estimated from each CSF samples collected from each suspected patient.

As the same time 3 ml venous blood samples were also collected in sterile containers by trained laboratory technologist and transported to the laboratory within half an hour after collection to perform total White blood cell, Red blood cell, Platelet, and differential cell count and determination of Hgb, HCT, MCV, MCH and MCHC using hematological auto-analyzer (Ruby Cell-Dyne 3000 USA).

### Quality control

The quality of socio-demographic and clinical data was maintained by using a structured questionnaire. The quality of culture media was checked by using reference strains and 10% of the prepared media before inoculation were checked by incubating at 35–37 °C for 24 h and observing the growth of an organism. The quality of reagents and antibiotics were maintained and used based on the manufacturer’s instruction and standard operating procedure (SOP) in place.

### Data analysis

The collected data were checked for completeness and consistency, next entered and analyzed using SPSS version 20 (IBM Corp., Armonk, NY, USA). Descriptive statistics such as frequency, percentage, and cross-tabulation were used to present the findings. A chi-square test was performed to evaluate the presence of a statistically significant association. The association between variables was assessed using the crude odds ratio (COR) from a binary logistic regression analysis. Adjusted odds’ ratio (AOR) was also computed using multivariable logistic regression analysis, taking all factors yielding a *p*-value ≤0.2 in bivariate analysis. A *P*-value of less than 0.05 was considered as statistically significant.

## Data Availability

The datasets used and/or analyzed during the current study are available from the corresponding author upon reasonable request.

## Abbreviations

AFB: Acid Fast Bacilli
AMS: Antimicrobial Susceptibility
BM: Bacterial Meningitis
CSF: Cerebrospinal Fluid
HIV: Human immunodeficiency virus
LP: Lumbar Puncture
SNNPR: Southern Nations and Nationalities People’s Region
WHO: World Health Organization
