# Correction to: Antimicrobial sensitivity profile and bacterial isolates among suspected pyogenic meningitis patients attending at Hawassa University Hospital: Cross-sectional study

**DOI:** 10.1186/s12866-020-01846-z

**Published:** 2020-06-11

**Authors:** Demissie Assegu Fenta, Kinfe Lemma, Henok Tadele, Birkneh Tilahun Tadesse, Birrie Derese

**Affiliations:** 1 School of Medical Laboratory Science, Hawassa University College of Medicine and Health Sciences, Hawassa, Ethiopia; 2 Department of Internal Medicine, Hawassa University College of Medicine and Health Sciences, Hawassa, Ethiopia; 3 Department of Pediatrics, Hawassa University College of Medicine and Health Sciences, Hawassa, Ethiopia; 4 Department of Neurology, Hawassa University College of Medicine and Health Sciences, Hawassa, Ethiopia

**Correction to: BMC Microbiology (2020) 20:125**

**https://doi.org/10.1186/s12866-020-01808-5**

Following publication of the original article [1], we have been notified that the tagging of one of the author names was done incorrectly. Original and corrected tagging can be seen below. The original article has been corrected.

- Incorrect name tagging:
  Last Name: Tadesse Tilahun
  First Name: Birkneh
- Correct author name tagging:
  Last Name: Tadesse
  First Name: Birkneh Tilahun
